# A new species of *Halicyclops* (Copepoda, Cyclopoida, Cyclopidae) from a lagoon system of the Caribbean coast of Colombia

**DOI:** 10.3897/zookeys.459.7972

**Published:** 2014-12-01

**Authors:** Eduardo Suárez-Morales, Juan M. Fuentes-Reinés

**Affiliations:** 1 El Colegio de la Frontera Sur, Unidad Chetumal, A.P. 424, 77014 Chetumal, Quintana Roo, México; 2 Universidad del Magdalena, Grupo de Investigación en Limnología Neotropical, A. A. 731 Santa Marta, Colombia

**Keywords:** Brackish waters, taxonomy, crustaceans, halicyclopines, lagoon systems biota

## Abstract

Plankton samples obtained from the lagoon system Laguna Navío Quebrado, in northern Colombia, yielded male and female specimens of an undescribed cyclopoid copepod of the genus *Halicyclops*. The new species belongs to the highly diverse and widely distributed *thermophilus*-complex. It closely resembles *Halicyclops clarkei* Herbst, 1982 from Louisiana and *Halicyclops bowmani* Rocha & Iliffe, 1993 from Bermuda. These species share the same armature of P1-P4EXP3, with a 3443 spine formula and the terminal antennary segment with 5 setae. However, *Halicyclops gaviriai* **sp. n.** can be separated from both *Halicyclops clarkei* and *Halicyclops bowmani* by the morphology of the anal pseudoperculum, the proportions of the fourth antennulary segment, the length of the inner basipodal spine of P1, the P1EXP/inner basipodal spine inner length ratio and the length/width ratio of the caudal rami. This is the third species of *Halicyclops* recorded from Colombia and the first one described from this country. With the addition of *Halicyclops gaviriai* **sp. n.**, the number of species of *Halicyclops* known from the Neotropics increases to 19. The regional diversity of the genus is probably underestimated.

## Introduction

The cyclopoid copepod genus *Halicyclops* is the most speciose in the subfamily Halicyclopinae; currently, it is known to contain 111 species and subspecies (Boxshall 2014) and is in need of revision. Members of this genus are cosmopolitan and planktonic forms (Chang 2012; Ueda and Nagai 2012), inhabiting chiefly coastal brackish water habitats, but some species can be found in freshwater habitats (Rocha 1995; Bazilevich and Kaftannikova 1970; Defaye and Dussart 1988; Fuentes-Reinés and Zoppi 2013).

In the Americas, Brazil and the United States are the countries with most records of *Halicyclops* (Wilson 1958; Herbst 1977, 1982; Rocha 1983, 1984, 1991, 1995, Rocha and Hakenkamp 1993). According to Rocha et al. (1998) there are about 17 species of *Halicyclops* recorded in the Caribbean region and this figure remained stable until the recent description of a new species from Argentina (Menu-Marque and Sorarrain 2007). In Colombia, the knowledge about this genus is still very limited; up to now, only two species, *Halicyclops venezuelaensis* Lindberg, 1954 and *Halicyclops exiguus* Kiefer, 1934 have been reported from two Caribbean localities of Colombia: Ciénaga Grande de Santa Marta, Magdalena and Laguna Navío Quebrado, La Guajira, respectively (Fuentes-Reinés et al. 2013, Fuentes-Reinés and Suárez-Morales unpubl. data). The few reports of *Halicyclops* in Colombia together with the high potential diversity of the genus in the area emphasizes the importance and necessity of intensifying the biological research in fresh and brackish water body in the country to improve our knowledge about the copepod fauna living in these environments. During a survey of the plankton community of the lagoonal system of Laguna Navío Quebrado, in the Colombian coast of the Caribbean, male and female specimens of an undescribed species of *Halicyclops* were collected. The aim of this paper is to describe this new species and compare it with its closest congeners.

## Methods

Plankton samples were taken monthly from the Laguna Navío Quebrado, Colombia (11°25'N, 73°5'W) between April and December 2012, mainly in the littoral areas with vegetation (macrophytes and mangrove) but also from open water in areas close to oyster banks. Water salinity was measured with a WTW 3111 conductivity meter. Water samples were collected using a bucket of 25 L at both vegetation areas and shallow open water. Samples were filtered with a zooplankton net (45 μm) and preserved in 70% ethanol. Copepods were sorted from the original samples and then processed for taxonomical identification. Dissected specimens and appendages were mounted in glycerine and sealed with Canada balsam. Drawings were made with the aid of a camera lucida mounted on an Olympus BX51 compound microscope equipped with Nomarski DIC. The specimens were measured in lateral position, from the anterior end of the rostral area to the posterior margin of the caudal ramus. The specimens examined were deposited at the Museo de Colecciones Biológicas at the Universidad del Atlántico (UARC), Colombia and in the Collection of Zooplankton (ECO-CHZ) held at El Colegio de la Frontera Sur (ECOSUR), Chetumal, Mexico. Morphological terminology follows Huys and Boxshall (1991). The following abbreviations are used in the description: P1–P6= first to sixth swimming legs, EXP= exopod, ENP= endopod.

## Results

### Taxonomy Order Cyclopoida Burmeister, 1834 Family Cyclopidae Dana, 1846 Subfamily Halicyclopinae Kiefer, 1927 Genus *Halicyclops* Norman, 1903

#### Halicyclops gaviriai sp. n.

Taxon classificationAnimaliaCyclopoidaCyclopidae

http://zoobank.org/6F89C13E-501E-4CAE-82FA-89D435118FCD

##### Material examined.

Adult female holotype (UARC393Z), Laguna Navío Quebrado, Colombia, limnetic plankton sample, 7 N., 2007, coll. Juan M. Fuentes-Reinés. Male allotype (UARC394Z), both partially dissected. Paratypes: ten females and four males, undissected, ethanol-preserved, vial (UARC395Z), plus one dissected female, slides (UARC399Z-403Z) and one dissected male (UARC397Z). Three adult females from same locality and date, two of them undissected, ethanol-preserved, in vial, one mounted on slide (ECO-CHZ-09267).

##### Type locality.

Laguna Navío Quebrado, La Guajira, northern Colombia (11°25'N; 73°5'W).

##### Description of female.

**Figure 1.**
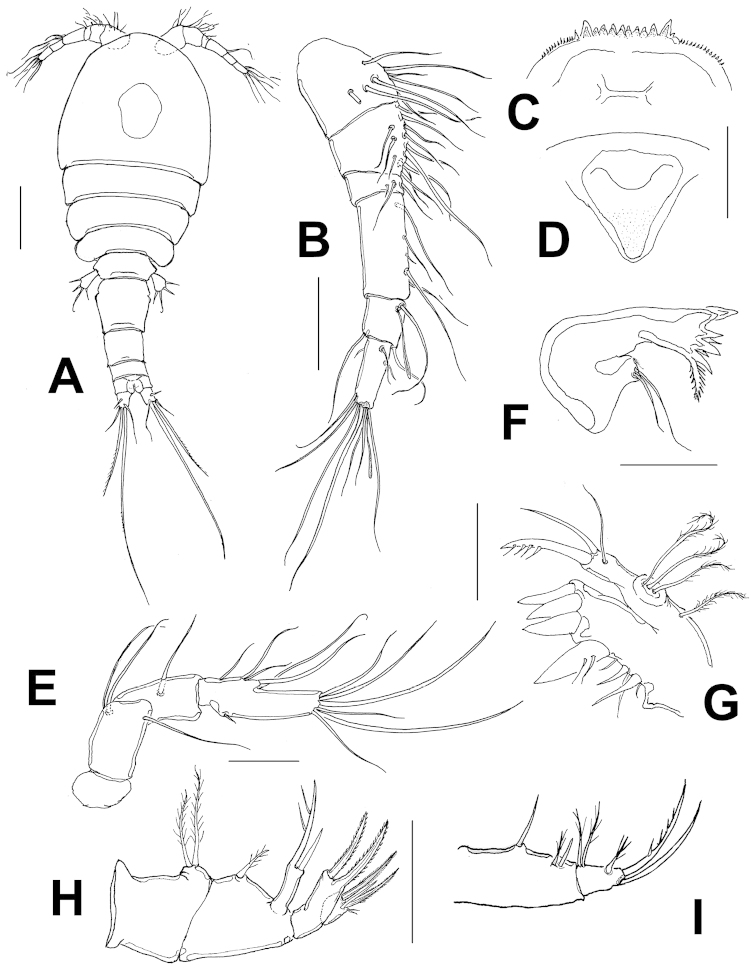
*Halicyclops gaviriai* sp. n., adult paratype female from northern Colombia. **A** habitus, dorsal view **B** antennule **C** labrum, ventral **D** rostrum **E** antenna **F** mandible **G** maxillule **H** maxilla **I** maxilliped. Scale bars: **A**=100 μm, **B–F**= 50 μm.

Habitus in dorsal position as in Figure 1A, body wide, robust in the anterior part, rostrum subtriangular. Body length, excluding caudal setae, 560–602 μm (average = 581 μm; *n*= 10; holotype: 574 μm). Rostrum strong, subtriangular (Fig. 1D). Labrum represented by widely rounded fig ornamented with marginal rows of spinules at both sides of teeth. Labrum armed with 10–12 teeth of different sizes, outermost being largest (Fig. 1C).

Cephalosome with large rounded dorsal integumental window. Urosome with four segments, genital double somite as long as wide with slight lateral protrusion at halflength and rounded integumental window on each side of posterior half (Fig. 3A). Seminal receptacle as shown in Fig. 3C, anal pseudoperculum, formed by slightly curved expansion of hyaline frill, posterior margin with irregularly serrate pattern (Fig. 3B) with adjacent rows of minute spinules. Caudal ramus about 1.2 as long as wide, outer seta III 1.4 times as long as ramus, apical seta V about twice time as long as seta IV (Fig. 1A), latter caudal seta with heteronomous ornamentation, with inner margin spinulated, outer margin with setules (Fig. 3D). Dorsal caudal seta (VII) 2.4 times as long as ramus.

Antennules 6-segmented, setal formula as follows, s=setae, ae=aesthetasc: 1(8s), 2(12s), 3(3s), 4(5s), 5(3+ae), 6(10+ae); fourth segment about 1.7 times as long as wide (Fig. 1B).

Antenna consisting of 4 segments, coxa reduced and unarmed, basis with 2 setae at inner corner; seta representing EXP present. ENP two-segmented. Proximal endopodal segment with a seta on middle inner margin. Terminal endopodal segment about 1.4 times as long as preceding segment armed with 5 inner setae and 7 apical setae plus short spinule on proximal outer margin. Length/wide ratio of second segment about 2.3 (Fig. 1E).

Mandible with well-developed coxal gnathobase, armed with 7 teeth plus outermost dorsal pinnate seta. Palp reduced, represented by 2 naked setae inserted on small protuberance, one seta about 1/3 times as long as the other one (Fig. 1F)

Maxillule with praecoxal arthrite bearing four strong tooth-like spines distally, inner spine strongest, with two proximal subequal setae, inner surface with two robust setal elements and one regular seta. Palp two-segmented, basis with 4 setae, endopodite represented by single oval-shaped segment, armed with three subequal, lightly setulated setae (Fig. 1G).

Maxilla 4-segmented, comprising praecoxa, coxa, basis and 1-segmented endopod. Praecoxal endite robust, armed with 3 setae and 2 spiniform elements on inner margin, with distal set of four robust claw-like spines. Basis with three elements including a claw-like spine, one naked stout seta and a short slender seta, exopod represented by single proximal seta. Endopod with 3 setae (Fig. 1H).

Maxilliped 2-segmented, armed with 3 setal elements on basal segment and 5 setae on distal segment, one of them subdistal, two distal (Fig. 1I).

**Figure 2.**
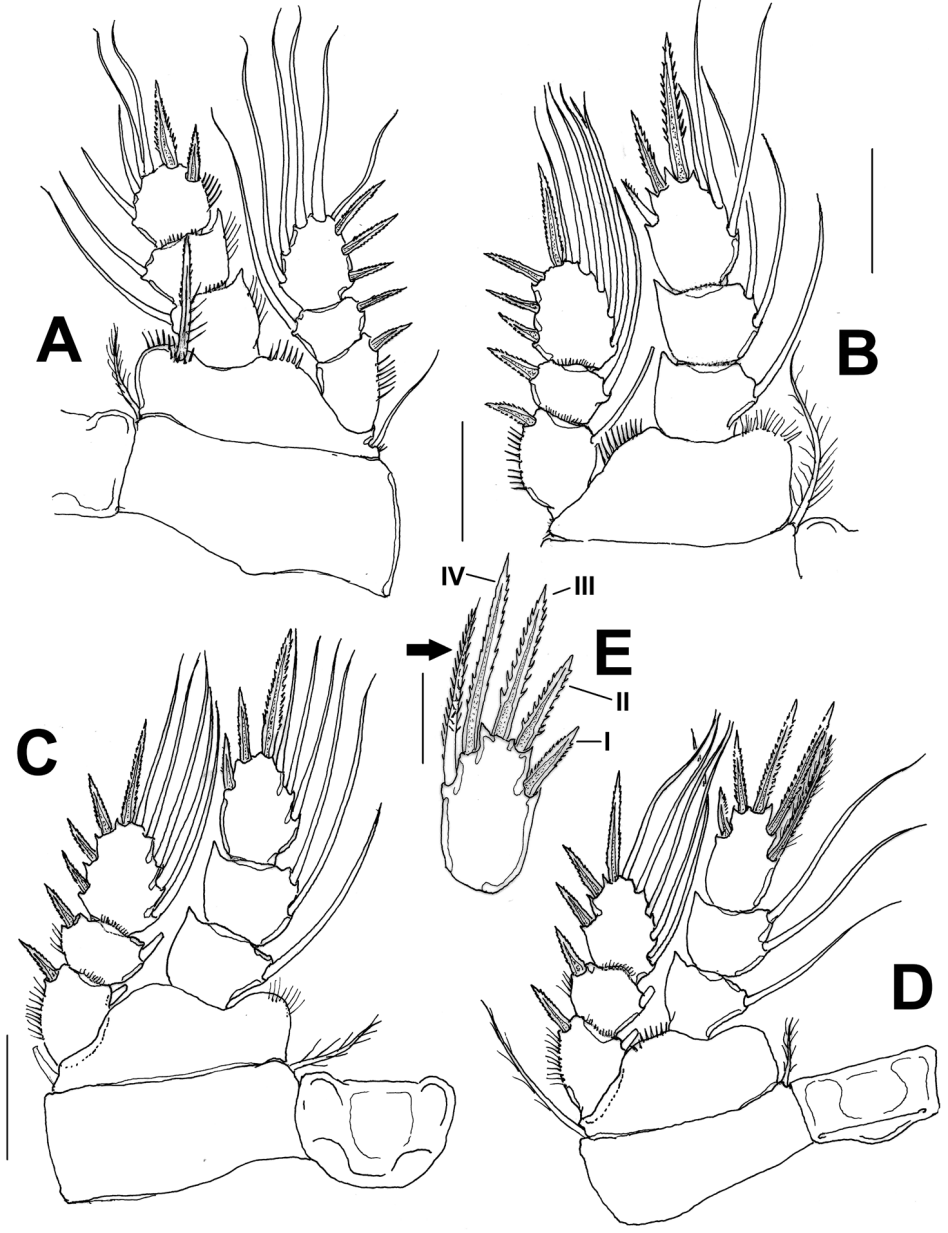
*Halicyclops gaviriai* sp. n., adult holotype female from northern Colombia. **A** leg 1 **B** leg 2 **C** leg 3 **D** leg 4 **E.** leg 4 terminal endopodal segment showing details of armature. Scale bars: **A–D**= 50 μm, **E**=25 μm.

P1-P4 exopod and endopod 3-segmented (Fig. 2A–D), armed as in Table 1. Spine inserted at inner corner of P1 basis reaching distal margin of second endopodal segment of P1 (Fig. 2A). EXP/inner spines of P1 basis ratio = 1.63. P2–P3 similar each other (Fig. 2B, C). Outer basipodal seta present in P1, P3 and P4, absent in P2. P4ENP3 about 1.7 times as long as wide, with four pinnate spines (I-IV) and inner lateral seta (arrowed in Fig. 2E), inner apical spine (III in Fig. 2E) as long as segment and 1.4 times as long as outer apical spine (II). Inner lateral spine (IV) 1.5 times as long as segment. Inner lateral seta spiniform, ornamented with short stiff setules.

**Figure 3.**
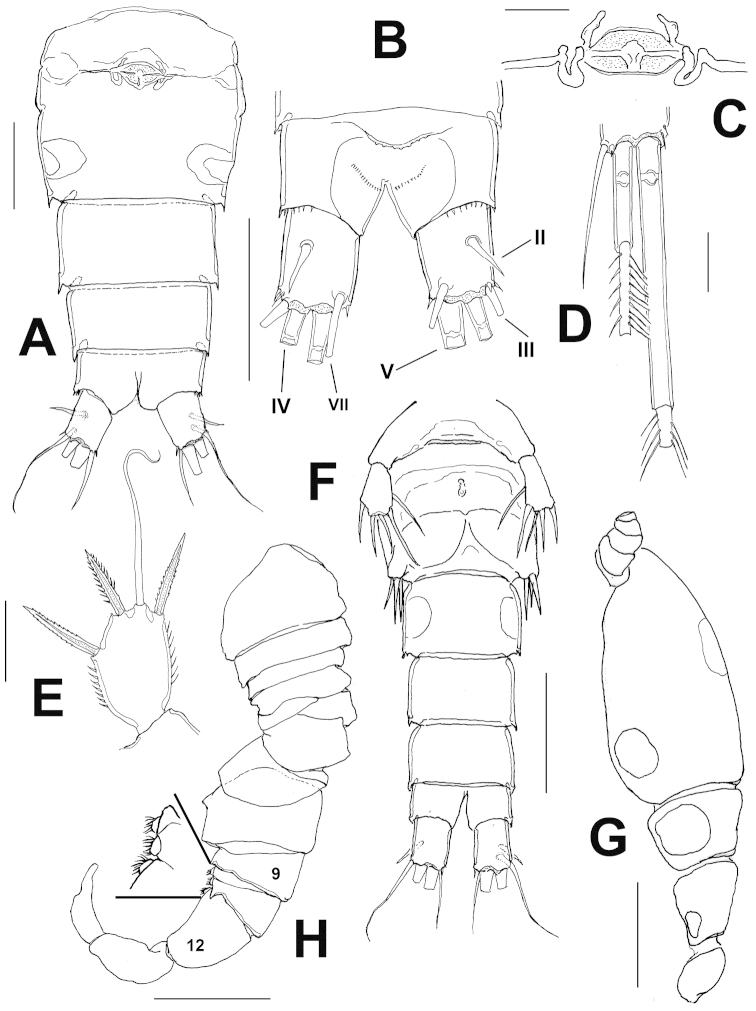
*Halicyclops gaviriai* sp. n., adult holotype female from northern Colombia. **A** urosome showing genital somite, ventral view **B** anal somite showing anal pseudoperculum, dorsal view **C** internal structures of genitalia, ventral view, **D** proximal section of middle apical setae of caudal ramus **E** leg 5; adult male from same locality **F** urosome, ventral view **G** lateral view of cephalothorax showing position of integumental windows **H** geniculate antennule, showing brush-like modified setae on segments 10–12. Scale bars: **A,B, F, H** =50 μm, **C–E**= 25 μm, **G**=100 μm.

P5 exopod subrectangular (Fig. 3E), about 1.56 times as long as wide, armed with 3 spines, all of them shorter than segment, plus one flexible seta 1.2 times as long as segment; relative length of elements from inner to outer margin as follows 0.66, 1.0; 0.46; 0.6.

##### Description of male.

Habitus resembling that of female, body length, excluding caudal setae= 420µm; (average = 410 μm; n = 10; holotype: 420 μm). Cephalosome with middle integumental window dorsally and lateral window on posterior margin. Second and third somites of prosome with integumental windows laterally, the latter being smallest (Fig. 3G). Rostrum as in female, antennules geniculate, 14-segmented (Fig. 3H), antennular segments 10-12 with modified brush-like setae (detail in Fig. 3H). Antenna, maxilla, maxillule, mandible and maxilliped as in female. Urosome with six somites, third somite with integumental window dorsally (Fig. 3F), caudal rami as in female.

**Figure 4.**
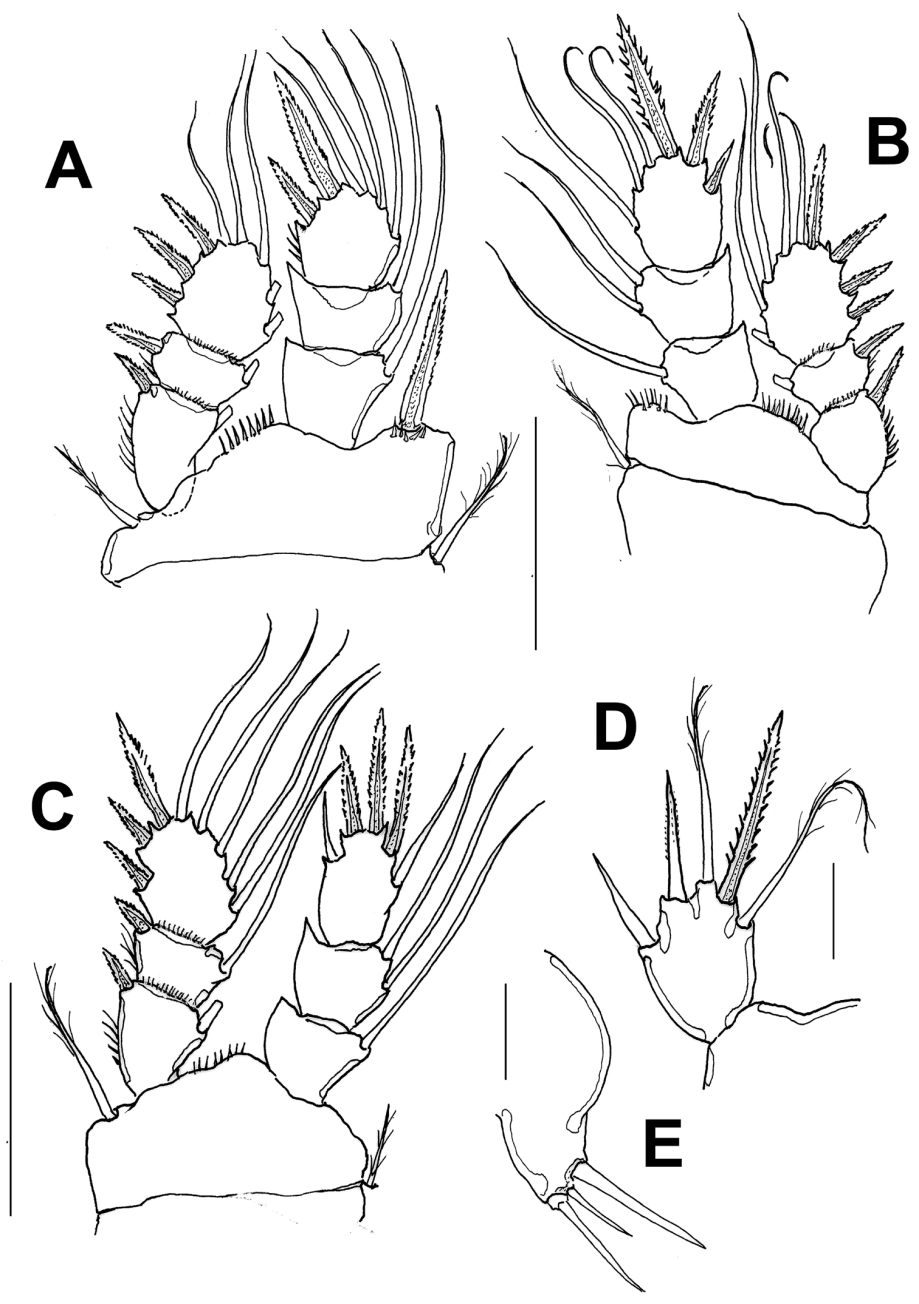
*Halicyclops gaviriai* sp. n., adult male allotype from northern Colombia. **A** leg 1 **B** leg 2 **C** leg 4 **D** leg 5 **E** leg 6. Scale bars: **A–C**= 50 μm, **D**=25 μm, **E**= 10 μm.

P1-P4 as in female (Fig. 4A–C), P5 exopod subrectangular, about 1.27 as long as wide, and bearing 3 spines and 2 setae (Fig. 4D), relative length of elements from inner to outer margin as follows 1.0, 0.8, 1.0; 0.6, 0.5. Sixth leg represented by fig with three elements, two stout setae, middle seta shortest (Fig. 4E).

##### Etymology.

The species is named after Dr. Santiago Gaviria for his work on Colombian copepods and his leadership in the formation of new generations of planktologists.

##### Remarks.

*Halicyclops gaviriai* sp. n. is assigned to the group of species “B” of *Halicyclops* with a 3443 spine formula; this is the most diverse group containing 74 species (see Pesce 2014). One of its subgroups, including approximately 15 species (Pesce 2014) is the *thermophilus*- complex, proposed by Herbst (1983). Species in this group share the presence of a chitinous blunt hook-like process on each side of the genital double-somite, but in *Halicyclops gaviriai* this process is reduced or absent. Other characters related to this group include: inner distal margin of the basis of leg 1 devoid of setae, thus diverging from *Halicyclops gaviriai* sp. n. with a well-developed inner basipodal spine. Two characters of the *thermophilus* group present in our specimens are: intercoxal sclerite of P1-P4 naked, and regular, unmodified setae on P4 EXP2-3. Because of the absence of the main group characters, the new species is not assigned to the *thermophilus*-complex. In Colombia, only one species of the *thermophilus* group has been hitherto recorded: *Halicyclops venezuelaensis* Lindberg, 1954.

Among the species of *Halicyclops* reported from the Caribbean region and adjacent areas (Rocha et al. 1998), *Halicyclops gaviriai* sp. n. closely resembles *Halicyclops clarkei* Herbst, 1982 described from Louisiana and *Halicyclops bowmani* Rocha & Iliffe, 1993 from Bermuda. Both of them lack strong processes on the genital double-somite and have a P1 with a strong inner basipodal spine (Herbst 1982; Rocha and Iliffe 1993; Pesce 2014). When the most recent key to the Neotropical species of *Halicyclops* (Rocha et al. 1998) is followed, our specimens from Colombia key down to a couplet leading to these two species (*Halicyclops clarkei*, *Halicyclops bowmani*). They share the same spine formula of P1-P4EXP3 (3443), the P4EXP3 with 3 spines on the outer margin, and the terminal antennulary segment with 5 lateral setae. The female fifth legs of these species are also very similar (Herbst 1982; Rocha 1991). However, *Halicyclops gaviriai* sp. n. can be separated from both *Halicyclops clarkei* and *Halicyclops bowmani* by differences in several characters. In *Halicyclops clarkei* the integumental windows of the genital double-somite are rounded and relatively small (Herbst 1982, fig. 15) whereas they are oblong and larger in the new species (Fig. 3A). The morphology and ornamentation of the anal pseudoperculum has been regarded of taxonomical value to distinguish species in this group (Rocha and Iliffe 1993; Pesce 2014). This structure is slightly curved and bears tiny denticles along the free margin in *Halicyclops clarkei* (Herbst 1982, fig. 16), it is strongly developed and coarsely serrate in *Halicyclops bowmani* (Rocha and Illife 1993, fig. 27), and it has shallow, irregular indentations, and is slightly curved in the new species (Fig. 3B).

The length/width ratio of the fourth antennulary segment differs in these species, it is much shorter in *Halicyclops gaviriai* (ratio = 1.7) *vs.* 2.5 in *Halicyclops bowmani* (Rocha and Iliffe 1993) and 2.7 in *Halicyclops clarkei* (Herbst 1982, fig. 18). Also, in *Halicyclops clarkei* the inner basipodal spine of P1 is long, slender, it reaches half of P1ENP3 (Herbst 1982, fig. 19), in *Halicyclops bowmani* this spine is more robust and shorter, it doesn’t reach the distal margin of P1ENP2 (Rocha and Illife 1993, fig. 29), whereas in *Halicyclops gaviriai* this spine reaches the distal margin of P1ENP2 (Fig. 2A). The length ratio P1EXP/basipodal spine is about 2.0 in *Halicyclops bowmani* (Rocha and Iliffe 1993, fig. 29), 1.42 in *Halicyclops clarkei* (Herbst 1982, fig. 19), and 1.63 in *Halicyclops gaviriai*.

The armature details of P4ENP3 shows some additional differences among these species; this segment is armed with 4 spines and one spiniform, distally serrate seta in both *Halicyclops clarkei* (Herbst 1982, fig. 22; Rocha 1991, fig. 10) and *Halicyclops gaviriai* sp. n., while in *Halicyclops bowmani* the armature consists of 3 spines and 2 stout, plumose setae (Rocha and Iliffe 1993, fig. 31). Also, in *Halicyclops clarkei* the inner apical spine of P4ENP3 is as long as the segment (Herbst 1982, fig. 22; Rocha and Hakenkamp 1993), whereas in both our specimens from Colombia and in *Halicyclops bowmani* (Rocha and Iliffe, 1993, fig. 31) this spine is 1.25 times as long as the segment (Fig. 2D, E). The proportions of the caudal ramus have some variation among these species, the length/width ratio is about1.5 in *Halicyclops clarkei*, 1.3 in *Halicyclops bowmani*, and 1.2 in *Halicyclops gaviriai* sp. n. The inner/outer apical caudal setae length ratio is 1.8 in both the new species and in *Halicyclops bowmani* (Rocha and Iliffe 1993, fig. 28) *vs.* 2.3 in *Halicyclops clarkei* (Herbst 1982, fig. 14). The body size of these species show some additional differences: measuring 560-602 μm, the female of the new species *Halicyclops gaviriai* is larger than those of *Halicyclops bowmani* (500-530 μm) (Rocha and Iliffe 1993), but smaller than the females of *Halicyclops clarkei* (698 μm) (Herbst 1982).

The new species has also affinities with Halicyclops cf. clarkei from Panama (Rocha 1991), but can be easily distinguished from the new species from Colombia by the armature of the female P5, in Halicyclops cf. clarkei the outermost spine is slightly shorter than the innermost and both are longer than the terminal segment (Rocha 1991, fig. 13), but in the new species the innermost spine is as long as the segment and the outermost spine is shorter than the segment (Fig. 3E). According to Rocha (1991), in both Halicyclops cf. clarkei from Panama and *Halicyclops clarkei* from the type locality in Louisiana the length/width ratio of the fourth antennulary segment are identical, about 2.7; this value diverges from that found in *Halicyclops gaviriai* (1.7). Also, in Halicyclops cf. clarkei the ENP3 of P2-P3 have the proximalmost inner seta modified as a stiff ornamented seta as the proximal seta of ENP3 of P4 (Rocha 1991, fig. 10), but in the new species these seta are unmodified, flexible elements (Fig. 2B, C). Rocha (1991) stated that the differences between the Panama specimens of Halicyclops cf. clarkei and those from the type locality in Louisiana are probably related to different species.

The male of the new species *Halicyclops gaviriai* differs from the male of *Halicyclops clarkei* in the presence of modified setae on the antennular segments 10-11, lacking in *Halicyclops clarkei* (Herbst 1982, fig. 25). Also, the length/width ratio of P4ENP4 is about 1.63 times as long as wide in *Halicyclops gaviriai* sp. n., while in *Halicyclops clarkei* is 1.53. The length/width ratio of P5EXP is about 1.27 in *Halicyclops gaviriai* sp. n., vs. 1.64 in *Halicyclops clarkei* (Herbst 1982, fig. 26). In *Halicyclops clarkei* the outer seta of P6 is clearly longer than the inner spine (Herbst 1982, fig. 27), whereas in *Halicyclops gaviriai* sp. n., the opposite condition occurs, the outer seta is shorter. Unfortunately, the male of *Halicyclops bowmani* remains unknown (Rocha and Iliffe 1993) and could not be compared with the male of the new species.

*Halicyclops gaviriai* sp. n. is characterized by a unique combination of characters including: 1) last antennary segment with five lateral setae, 2) length/wide ratio of same segment over than twice as long as wide, 3) fourth segment of female antennule over than twice as long as wide, 4) inner basipodal spine of P1 reaching the posterior border of the ENP2 of P1, 5) ENP3 of P4 with four serrate spines and one seta, and 6) P5 about 1.45 times as long as wide, apical seta longer than the segment.

*Distribution and ecology. Halicyclops gaviriai* sp. n. is currently known from a single locality only, the protected coastal system Laguna Navío Quebrado, on the Caribbean coast of Colombia. This species was recorded in both the limnetic region and the vegetation zones, being more frequent in the former habitat. This large (surface area of 10.7 km^2^) lagoon system is a shallow water body (depth 0.3–1.1 m), whose temperature varies over the seasons in the range of 28–31 °C; pH values during sampling ranged between 7.8 and 8.3 and salinity was 28 PSU. This habitat diverges from that of one of its closest congeners, *Halicyclops bowmani*, a stygobitic form recorded only from an anchialine system of Bermuda (Rocha and Iliffe 1993). The known habitat of its other close congener, *Halicyclops clarkei*, is Lake Peigneur, a former freshwater system whose salinity drastically increased since 1980 after a failed oil drill deeply modified the system (Zio and Aven 2013). The samples examined by Herbst (1982) were obtained before this event, in 1977; he reported a low salinity range (0–5 psu) for this species. Hence, it is an intriguing question if this presumably endemic species was able to adapt to the new conditions and is still extant in the locality or adjacent areas.

The number of Neotropical species recognized by Rocha et al. (1998) was 17 and it remained stable in the region until the recent description of *Halicyclops ramirezi* from Argentina (Menu-Marque and Sorarrain, 2007) and the addition of this new species from Colombia, thus raising the number of known Neotropical species to 19. Furthermore, some nominal species in the literature such as Halicyclops cf. clarkei from Panama (Rocha 1991) probably represent undescribed species. The diversity of the genus in the region could be underestimated and certainly deserves further investigations.

**Table 1.** Armature formula of legs 1–4.

	coxa	basis	exopod	endopod
Leg 1	0-1	1-I	I-0, I-1,III-1,4	0-1,0-1,II-2,2
Leg 2	0-1	0-0	I-1,I-1,III-1-1,4	0-1,0-2, III-3
Leg 3	0-1	1-0	I-1,I-1,III-I-1,4	0-1,0-2, III-3
Leg 4	0-1	1-0	I-1, I-1, II-I1-4	0-1,0-2, I-II-II

## Supplementary Material

XML Treatment for Halicyclops gaviriai
